# A distress-continuum, disorder-threshold model of depression: a mixed-methods, latent class analysis study of slum-dwelling young men in Bangladesh

**DOI:** 10.1186/s12888-021-03259-2

**Published:** 2021-06-04

**Authors:** Syed Shabab Wahid, John Sandberg, Malabika Sarker, A. S. M. Easir Arafat, Arifur Rahman Apu, Atonu Rabbani, Uriyoán Colón-Ramos, Brandon A. Kohrt

**Affiliations:** 1 Department of Global Health, Milken Institute School of Public Health, George Washington University, 950 New Hampshire Ave NW #2, Washington, DC 20052 USA; 2 Department of Psychiatry and Behavioral Sciences, Division of Global Mental Health, George Washington University, 2120 L street NW, Suite 600, Washington, DC 20037 USA; 3 BRAC James P Grant School of Public Health, BRAC University, 5th Floor, (Level-6), icddr,b Building 68 Shahid Tajuddin Ahmed Sharani, Mohakhali, Dhaka, 1212 Bangladesh; 4 Heidelberg Institute of Global Health, Heidelberg University, Heidelberg, Germany; 5 Department of Economics, University of Dhaka, Dhaka, Bangladesh

**Keywords:** Depression, Classification, LCA, Mixed methods, LMIC, Slum, Bangladesh

## Abstract

**Background:**

Binary categorical approaches to diagnosing depression have been widely criticized due to clinical limitations and potential negative consequences. In place of such categorical models of depression, a ‘staged model’ has recently been proposed to classify populations into four tiers according to severity of symptoms: ‘Wellness;’ ‘Distress;’ ‘Disorder;’ and ‘Refractory.’ However, empirical approaches to deriving this model are limited, especially with populations in low- and middle-income countries.

**Methods:**

A mixed-methods study using latent class analysis (LCA) was conducted to empirically test non-binary models to determine the application of LCA to derive the ‘staged model’ of depression. The study population was 18 to 29-year-old men (*n* = 824) from an urban slum of Bangladesh, a low resource country in South Asia. Subsequently, qualitative interviews (*n* = 60) were conducted with members of each latent class to understand experiential differences among class members.

**Results:**

The LCA derived 3 latent classes: (1) Severely distressed (*n* = 211), (2) Distressed (*n* = 329), and (3) Wellness (*n* = 284). Across the classes, some symptoms followed a continuum of severity: ‘levels of strain’, ‘difficulty making decisions’, and ‘inability to overcome difficulties.’ However, more severe symptoms such as ‘anhedonia’, ‘concentration issues’, and ‘inability to face problems’ only emerged in the severely distressed class. Qualitatively, groups were distinguished by severity of *tension*, a local idiom of distress.

**Conclusions:**

The results indicate that LCA can be a useful empirical tool to inform the ‘staged model’ of depression. In the findings, a subset of distress symptoms was continuously distributed, but other acute symptoms were only present in the class with the highest distress severity. This suggests a distress-continuum, disorder-threshold model of depression, wherein a constellation of impairing symptoms emerge together after exceeding a high level of distress, i.e., a tipping point of *tension* heralds a host of depression symptoms.

## Background

In recent years, there is increasing critique of categorical models of depression wherein an individual either has or does not have the condition, and instead, there are calls for continuous and other approaches to conceptualizing depression [1–3]. The categorical approach, however, continues to be the hallmark of current diagnostic systems of psychiatry, namely the Diagnostic and Statistical Manual of Mental Disorders (DSM-5) and the International Classification of Disease [4]. In this binary categorical approach, symptom counts are used to diagnose the presence of depression: i.e., 4 out of 9 hallmark depression symptoms do not count as a diagnosis, but 5 out of 9 meet clinical criteria. However, growing research on depression indicates it to be continuously distributed in the general population along a continuum of severity [5–7]. Biomarkers associated with depression (e.g., inflammatory markers or cortisol) also show distributions within populations, rather than binary conditions where a biomarker is either present or absent [8].

A binary categorical approach has clinical limitations and potential negative consequences. Using symptom thresholds for diagnosis increase the possibility of false positives [9], and symptom equivalences may mischaracterize the disorder, e.g., a change in appetite and suicidality are weighted equally when meeting diagnostic criteria [10]. Relying on a binary approach to diagnosis can also lead to inflated prevalence rates and overestimation of disease burden [9]. Affixing otherwise healthy people with a diagnosis of mental illness also makes them vulnerable to societal stigma [11]. For those who do get connected to services, there is potential for a mismatch between condition and intervention, increasing the possibility of harm, especially if later-stage interventions are incorrectly initiated earlier [12].

Acknowledging these shortcomings, the National Institute of Mental Health proposed reconceptualizing the processes underlying mental disorders according to dimensions (negative and positive valence systems; cognitive systems; social processes systems; and arousal/modulatory systems) across developmental trajectories and environmental effects [4]. However, this dimensional approach has been criticized by clinicians due to its complexity and apparent lower clinical utility in diagnostic decision-making [5].

One proposed solution that has clinical and public health utility, and also incorporates a continuum of distress, is to consolidate a categorical model into an ordinal one, organized from wellness, through to distress, and disorder, according to increasing severity or chronicity [5]. This hybrid ‘staged model of depression’ aligns with previous calls for the ‘staging’ of psychiatric disorders along the continuum of disease progression [12]. The proposed stages are: (1) Wellness – defined by the absence of any sustained distressful emotions or experiences; (2) Distress – defined by the presence of mild to moderate distressful emotional experiences; (3) Depressive disorder – defined by the presence of severely distressful experiences lasting 2–4 weeks with impairment of daily functioning; and (4) Refractory – defined by unresponsive or relapsing depression. The model provides a responsive solution to the heterogeneity of depressive symptoms by recommending specific interventions at each stage and corresponding platforms of delivery. This is beneficial as it shifts away from a ‘one-size-fits-all’ approach that can risk under- or over-treating individuals [5].

*The Lancet Commission for Global Mental Health and Sustainable Development* has recently incorporated the staged model as a key recommendation for the sustainable development goals [13]. The challenge of categorical approaches is especially important in low- and middle-income countries (LMICs) because of the risk of creating a ‘category fallacy’, i.e., medicalizing the normal range of negative affective experiences, as varying by culture [14]. Categorical cut-offs from one country applied to another country can incorrectly inflate disease burdens, which in turn, can overwhelm mental health care services if individuals are inappropriately referred to providers [15]. This is especially relevant when considering the low service capacity of depleted mental health systems in LMICs [16]. Using a staged model to link individuals with corresponding services (e.g., mental health promotion, community and self-care, primary health care, specialist care) would alleviate the burden on the limited capacity of specialist care platforms [5].

A potential statistical approach to informing the staged model is Latent Class Analysis (LCA), a method for classifying populations into groups based on observed characteristics. LCA has been used to inform various staged approaches in health, including stages of smoking cessation; stages of alcohol use; and risk factors of disease during developmental transitions [17–19]. Previously, LCA has been used in psychiatric research to empirically define sub-types of depression which found most LCA derived sub-groups based on depressive measures to be organized along a continuum of severity [20–25]. As LCA derives latent groups from the particular population under study, it incorporates depression variability across cultures (i.e., it does not rely on categorical instrument cut-offs that may not appropriate across cultures and populations). These multiple factors make LCA a potentially suitable candidate for informing the staged model of depression for global populations.

Therefore, in this study, we employ LCA to inform the staged model of depression in a population of slum-dwelling young men in Bangladesh, an LMIC in South Asia. In addition to LCA, we also included a qualitative component to account for criticism that LCA derived classes may not represent meaningful categories [26]. The specific objectives were to: (1) use LCA to classify the population into subgroups, according to self-reported depressive symptomology; and (2) utilize qualitative methods to understand experiential differences among LCA derived sub-groups.

## Methods

### Setting and population

This study was conducted with a group rarely included in mental health research: young men from an LMIC in a vulnerable urban environment. Slums of Bangladesh are rife with risk factors for depression such as persistent poverty, insecure housing and land tenure, poor sanitation, environmental hazards, legal disempowerment, crime, infectious and chronic disease, and injuries [27]. The focus on young men, in particular, is important as later adolescence and young adulthood represents a complex stage of development when most psychiatric illnesses like depression first manifest [28]. The location of the study was *Bhasantek*, a large impoverished urban slum home to mostly day laborers [29].

### Study design

This study used quantitative methods (LCA) to assign the population into groups based on their responses to a depression screening questionnaire. Afterwards, qualitative interviews were conducted with members of these groups to gain deeper understanding of their experience of distress and depression. We utilized a classic mixed-methods sequential explanatory design, where quantitative research is first conducted, followed by qualitative research to elaborate and explain the quantitative findings [30].

### Ethical approval

Ethical approval for this study was provided by the institutional review board of the BRAC James P. Grant School of Public Health, BRAC University. All research activities related to human subject participants were performed in accordance with the Declaration of Helsinki and approved by the ethics review board of BRAC University.

### Quantitative: sampling, data collection, and variables

The quantitative data for this study comes from a survey that was conducted as part of a larger project, focused on masculine gender norms and risky sexual behaviors [31]. Data was collected from a non-representative, cross-sectional community sample of 824 young men (ages 18–29), living in one division (out of four), in *Bhashantek* slum. A census survey was administered on-site in 2016 by trained enumerators covering all households in the study catchment area.

Depression was measured by the General Health Questionnaire-12 (GHQ-12) [32], a 12-item screening instrument used to determine non-psychotic, psychiatric morbidity. The GHQ-12 has been validated and widely used to screen for depression across various global populations [33–35], and in Bangladesh in both clinical and non-clinical settings [36–39].

### Statistical analysis

We conducted an empirical investigation of heterogeneity using LCA to classify the study population into latent sub-groups based on depressive symptoms. LCA classifies individuals from a heterogenous population into mutually exclusive and collectively exhaustive homogenous sub-groups based on the probability of class membership conditional on item response on a set of variables e.g., the items of the GHQ-12 [40]. LCA iteratively clusters similar responses using maximum likelihood to classify individuals into distinct classes. Therefore, individuals in each class are most similar to others in their class, and most distinct from individuals in other classes.

In this study, LCA was conducted on the items of the GHQ-12 questionnaire using MPlus software [41]. Using 300 random sets of starting values we evaluated a series of models incorporating 2 to 5 classes, with the addition of one extra class in each successive model. Model fit was assessed using the Akaike Information Criteria (AIC), Bayesian Information Criteria (BIC), the sample adjusted BIC (ABIC), and Entropy values, with smaller values of AIC, BIC, and ABIC, and Entropy values ≥0.8 indicating better model fit [42, 43]. We also used the Lo-Mendell-Rubin likelihood ratio test (LMR LRT) which compares improvement in fit between models (i.e., comparing *k* − 1 and the *k* class models) and generates a *p*-value to determine if there is a statistically significant improvement in model fit with the inclusion of additional classes [44].

GHQ-12 responses can be scored using a Likert style scoring (Never-0; Sometimes-1; Often-2; Always-3) or the classic (0–0–1-1) approach, that combines responses indicating frequent presence (Often & Always) of symptoms [45]. We used the Likert approach for the LCA analysis and, subsequently, the classic approach to investigate how means of GHQ-12 items tracked across each latent class.

### Qualitative: sampling and data collection

A stratified sampling strategy according to latent class membership (The LCA derived 3 classes; please refer to the results section) and symptom severity (as measured by the GHQ-12) was used to purposively recruit individuals for the explanatory qualitative portion of the study (Table 1) [46]. We oversampled for those latent classes with distressed or potentially disordered individuals, due to symptom heterogeneity.

**Table 1:** Sampling for qualitative phase stratified by latent class and symptom severity (GHQ-12 score)

		GHQ-12: 0–9	GHQ-12: 10–15	GHQ-12: > 15	Total
Latent Class 1	Available population	16	128	67	211
	**Qualitative sample**	**1**	**11**	**14**	**26**
Latent Class 2	Available population	109	220	0	329
	**Qualitative sample**	**12**	**11**	^a^	**23**
Latent Class 3	Available population	281	3	0	284
	**Qualitative sample**	**11**	^b^	^a^	**11**
				Total population	824
				**Total qualitative sample**	**60**

^a^ No individuals met the criteria in the population

^b^ Individuals were unavailable or did not consent to interview

A semi-structured interview guide was developed using categories of Kleinman’s explanatory model framework of mental disorders [47]. These included the cause, effects, severity and course, phenomenology and lived experience of distress and depression. The guide was piloted six times for comprehension and adjusted for cultural and contextual considerations, which included the incorporation of local idioms of distress [48]. Interviewers initiated inquiry on distress by eliciting the current mental and emotional state of the respondent and recent stressful life events. Using these experiences and events as references, the interview subsequently explored the explanatory models and signs and symptoms of distress and depression. We also explored respondents’ history and background and daily life in slums. The qualitative data was collected on-site in 2018 by two of the authors, both male anthropologists, who had intimate familiarity with the site and long-standing relationships with the participants. Interviews were conducted in respondents’ homes or private community spaces suggested by participants. The interviews were conducted in the local language, audio recorded with informed consent, and then professionally translated and transcribed into English.

### Qualitative analysis

The data from the qualitative interviews was analyzed by the first author, a native Bangla speaker with expertise in mixed methods research, in consultation with other authors. A codebook was iteratively developed using relevant theoretical literature and progressive addition of inductive codes, and subsequently used to code the dataset. We used NVivo, version 12, for data analysis [49]. We applied the constant comparison method, which involved moving back and forth between coded data and comparing it to previously coded segments, to ensure integrity of the content [50]. After coding was complete, we extracted code-specific data for each latent-class by executing code queries in NVivo stratified according to latent class membership. Subsequently, code summaries were written to capture insights specific to each latent class. We mixed the data by “embedding” qualitative results to contextualize the major quantitative findings, as conventionally done for sequential-explanatory mixed methods designs [30].

## Results

### Latent class analysis

A solution of three classes (‘Wellness;’ ‘Distressed;’ and ‘Severely distressed’) provided the best fit. The AIC, BIC and the ABIC for the 3-class model were 16,851.96, 17,370.52, and 17,021.2, respectively (Table 2). While the AIC, BIC, and ABIC continued to decrease, the LMR LRT lost statistical significance with the addition of a fourth and fifth class, indicating the 3-class model as the optimal solution. The 3-class model had an entropy value of 0.86 indicating clear delineation of classes [43]. The GHQ-12 had good internal consistency (Cronbach’s alpha coefficient = 0.83).

**Table 2:** Comparison of model fit for 2 to 5 latent classes

Latent class analysis: fit statistics				
	2 Class model	3 Class model	4 Class model	5 Class model
**AIC**	17,516.72	16,851.96	16,481.18	16,353.32
**BIC**	17,860.85	17,370.52	17,174.16	17,220.73
**ABIC**	17,629.03	17,021.2	16,707.34	16,636.41
**LMR LRT (p-value)**	0.000	0.000	0.7377	0.7672
**Entropy value**	0.88	0.86	0.85	0.86

### Sample characteristics

Demographic information of 824 male slum residents aged 18–29 years according to latent class membership is presented in Table 3. Latent class-1 had a higher percentage of respondents with no formal education (15.64%). The age of respondents was comparable across all three latent classes for all age groups. Latent class-1 had the highest percentage (40.28%) of respondents in the poorest wealth quintile. Class-1 reported the highest mean GHQ-12 score. The sample of respondents for the qualitative study is presented in Table 1.

**Table 3:** Demographic characteristics of respondents across latent classes

	Class 1	Class 2	Class 3	
	Severely distressed	Distressed	Wellness	
	(***n*** = 211)	(***n*** = 329)	(***n*** = 284)	χ^**2**^
Education (%)				24.16***
No formal education	15.64	6.69	9.86	
Up to 5th grade	35.07	52.89	52.11	
Higher than 5th grade	49.29	40.43	38.03	
Age (%)				8.96*
18–21 years	38.86	27.05	33.80	
22–25 years	30.81	34.35	31.34	
26–29 years	30.33	38.60	34.86	
Socioeconomic quintiles (%)				17.40**
Poorest	40.28	29.48	27.11	
Poorer	18.96	19.45	26.06	
Middle	10.90	19.45	16.55	
Richer	11.85	12,16	11.27	
Richest	18.01	19.45	18.93	
GHQ-12 Score (mean)	14.16***	10.48***	4.10***	

* *p* < 0.1; ** *p* < 0.05; *** *p* < 0.01

### Comparison of 3 latent classes

**Fig. 1:**
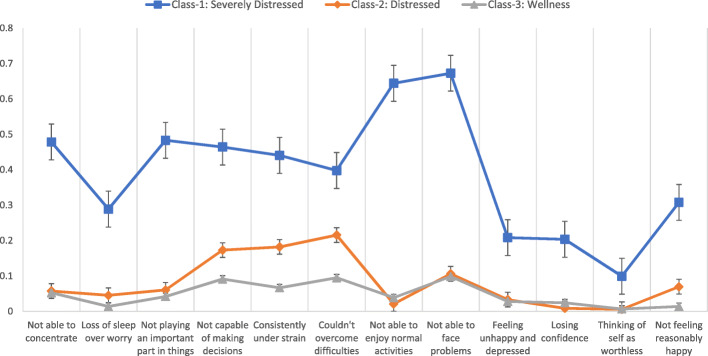
Means of dichotomized GHQ-12 items using classic scoring (0–0–1-1) across each latent class

In Fig. 1 we present the means of dichotomized GHQ-12 items using the classic scoring (0–0–1-1) approach. Latent classes 2 (Distressed) and 3 (Wellness) almost track in perfect alignment, except for the domains of strain, decision making and overcoming difficulties, which are elevated for the ‘distressed’ class. Hallmark symptoms of depression like anhedonia, loss of concentration and feelings of unhappiness emerge only for the ‘severely distressed’ class. Interestingly, ‘severely distressed’ class members do not report feelings of low self-worth, which are at comparable levels as the other classes. The qualitative research found pervasive masculine norms of projecting strength, and unwillingness to share emotions (“Why should someone else find out about my weaknesses?”), which provides context to this particular response pattern. In the following sections we present mixed quantitative and qualitative results starting with the Wellness group (Class 3) and progressing to higher levels of severity (Class 2 and 1). The observed class membership and endorsement frequencies for the GHQ-12 for this 3-class model are presented in Table X.

### Wellness: well-being and mildly stressed (latent Class-3)

There were 284 members (34.5%) in Class-3, with a GHQ-12 mean score of 4.09 (95% Confidence Interval [CI]: 3.79–4.40). Respondents in this class had the lowest endorsement across all GHQ symptoms (Table 4). These included the inability to enjoy normal activities (72%; ‘Never’); loss of sleep due to worry (67%; ‘Never’); concentration problems (72%; ‘Never’); indecisiveness (52%; ‘Never’); and depressed mood (76%; ‘Never’). Therefore, we labeled this class as “**Wellness**.” Even though this class had the highest response rates for the ‘Never’ category for stress related items, class members indicated some degree of difficulty facing problems (36%; ‘Sometimes’), being consistently under strain (39%; ‘Sometimes’), and trouble overcoming difficulties (28%; ‘Sometimes’). Most members emphatically endorsed never feeling worthless or losing self-confidence.

**Table 4:** Endorsement probabilities and class membership for GHQ-12 across latent classes

GHQ-12 Items	Severely distressed (*n* = 211)	Distressed (*n* = 329)	Wellness (*n* = 284)
**1. Not able to concentrate**			
Never	0.24	0.10	0.72
Sometimes	0.29	0.85	0.23
Often	0.44	0.06	0.05
Always	0.03	0.00	0.00
**2. Loss of sleep over worry**			
Never	0.22	0.33	0.67
Sometimes	0.50	0.62	0.31
Often	0.23	0.05	0.02
Always	0.05	0.00	0.00
**3. Not playing an important part**			
Never	0.15	0.16	0.79
Sometimes	0.38	0.78	0.17
Often	0.47	0.06	0.03
Always	0.01	0.00	0.01
**4. Not capable of making decisions**			
Never	0.10	0.03	0.52
Sometimes	0.43	0.81	0.39
Often	0.44	0.16	0.09
Always	0.03	0.00	0.00
**5. Consistently under strain**			
Never	0.07	0.20	0.54
Sometimes	0.50	0.62	0.39
Often	0.38	0.18	0.05
Always	0.05	0.00	0.03
**6. Couldn’t overcome difficulties**			
Never	0.01	0.03	0.63
Sometimes	0.59	0.76	0.28
Often	0.37	0.21	0.07
Always	0.03	0.00	0.03
**7. Not able to enjoy normal activities**			
Never	0.10	0.05	0.72
Sometimes	0.30	0.91	0.24
Often	0.59	0.03	0.03
Always	0.02	0.00	0.01
**8. Not able to face problems**			
Never	0.08	0.01	0.54
Sometimes	0.26	0.88	0.36
Often	0.64	0.11	0.09
Always	0.01	0.00	0.01
**9. Feeling unhappy and depressed**			
Never	0.34	0.31	0.76
Sometimes	0.45	0.66	0.21
Often	0.19	0.03	0.02
Always	0.02	0.00	0.01
**10. Losing confidence**			
Never	0.60	0.60	0.94
Sometimes	0.20	0.39	0.03
Often	0.09	0.01	0.01
Always	0.11	0.00	0.02
**11. Thinking of self as worthless**			
Never	0.68	0.58	0.95
Sometimes	0.22	0.41	0.04
Often	0.05	0.01	0.00
Always	0.05	0.00	0.00
**12. Not feeling reasonably happy**			
Never	0.40	0.17	0.82
Sometimes	0.29	0.77	0.16
Often	0.27	0.06	0.01
Always	0.03	0.01	0.01

Qualitative findings indicated that most “Wellness” class members attributed distress to financial burdens or relationship problems. However, these stressors were considered as mild, specific to certain events, and short-lived. When respondents in this class did refer to negative affective and cognitive mind states, they used the English word “*tension*,” a popular local idiom of distress. Most respondents reported being capable of handling life’s challenges, and either being free of *tension* or able to manage it. Financial stability, personal mental strength, and the presence of supportive relationships were discussed as reasons for well-being:

“When I’m sad or worried, it becomes difficult to fall asleep. I can’t fall asleep immediately as the thoughts go around in my head. *Tension* is something I consider a normal thing though. No one can be free of worries- it’s normal.” – Respondent-7 (GHQ-12 score: 6).

“No, no sadness. In childhood, I had my father, mother, siblings and relatives, and no problems with money for daily expenses. I am the youngest so never had any *tension*. Even now, if I don’t work for a year, I will get money from my village every month. No reason to feel sad - always happy with others.” – Respondent-20 (GHQ-12 score: 5).

### Distressed: under strain, indecisiveness, and can’t overcome difficulties (latent Class-2)

There were 329 individuals in Class-2 (39.9%) with a mean GHQ-12 score of 10.48 (CI: 10.25–10.71). This class had the highest endorsement for the “Sometimes” response category on all GHQ-12 items (Table 4). Class-2 endorsement frequencies indicated intermittent presence of depressive symptoms. These included the inability to enjoy normal activities (91%; ‘Sometimes’); loss of sleep due to worry (62%; ‘Sometimes’); concentration problems (85%; ‘Sometimes’); difficulty facing problems (88%; ‘Sometimes’); indecisiveness (81%; ‘Sometimes’); and depressed mood (66%; ‘Sometimes’). Accordingly, we labeled this class as “**Distressed**.” Like Class-3, most Class-2 members also did not report feeling worthlessness (58%) or lacking self-confidence (60%).

The qualitative interviews revealed that members of this class face substantial financial hardships and challenges over navigating social roles and expectations of assuming responsibility of their families. Most respondents’ experiences indicated some degree of affective distress and being under pressure that were tied to financial worries, concerns for family, or relationship problems with romantic partners or friends. As with the “Wellness” class, this group also used *tension* to convey distress. Unlike the “Wellness” class, this “Distressed” class reported having too much *tension*, unable to manage *tension*, and having consequences of *tension* such as insomnia:

“Every month I have to take loan of 4 or 5 thousand taka ($59 USD) to make ends meet … I am the only bread earner … my wife stays at home … with my earning I can’t maintain all costs … every month I have to borrow money … for that I have *tension* … I have to repay loans … how do I manage the rest of the month? This is how this year has been going … when I am in too much *tension* … say I took loan from you and its due tomorrow, but I couldn’t secure the money … because of that I have *tension* at night and lose sleep.” – Respondent-51 (GHQ-12 score: 9).

Overall, the quantitative results and qualitative descriptions did not invoke certain symptoms, some of which are in depression diagnostic criteria i.e., anhedonia, suicidality, and persistent low mood.

### Severely distressed: anhedonic, unable to face problems, and other depressive symptoms (latent Class-1)

Class-1 was comprised of 211 individuals representing 25.6% of the sample. The mean GHQ-12 score was 14.16 (CI: 13.67–14.65). Class-1 was characterized by the highest endorsement rates for persistent presence (‘Often’ and ‘Always’ categories of the GHQ-12 items) of DSM-5 depressive symptoms, compared to other classes (Table 4). Accordingly, we termed this class “**Severely distressed**.” These included the inability to enjoy normal activities (59%; ‘Often’), loss of sleep due to worry (23%; ‘Often’), difficulty facing problems (64%; ‘Often’), concentration problems (44%; ‘Often’), indecisiveness (44%; ‘Often’), and depressed mood (19%), indicating strong likelihood of underlying clinical psychiatric morbidity. Class-1 members also reported being under consistent strain and having trouble overcoming difficulties in life, suggesting a heavily burdened population under substantial stress. However, despite the presence of these symptoms, most members of this class did not report feeling worthless (68%; ‘Never’) or lacking self-confidence (60%; ‘Never’).

In the qualitative interviews, members of Class-1 reported living in conditions of amplified financial peril, working in unstable jobs or operating vulnerable street businesses. Respondents described challenges transitioning between adolescence and adulthood, with substantial demand from family to take on the role of the provider. Such social roles had a strong gender framing, with men reporting that they experienced substantial distress if they could not meet the expectations of this role. Relationship issues with romantic partners, family or friends were also attributed as major factors for distress. Some respondents reported specific circumstances like the death of a family member or personal illness or disability for their mental condition. Most Class-1 members reported experiencing persistent and severe distress, along with anhedonia, loss of sleep or appetite, feelings of worthlessness or persistent sadness, rumination and intrusive thoughts of self-harm or suicide, or actual self-harming and suicide attempts. One respondent shared:

“The police came without warning to bust up our shops. When they came no one could remain cheerful … my face became dark automatically … many kinds of *tension* were created: ‘What have I done? How do I pay rent? Pay family expenses? Run my business? Where do I get money to buy food tomorrow?’ Many kinds of thoughts came to the mind. When my shop got busted my mind got broken – it stopped working and stayed like that for a long time. I used to think ‘What to do?’ *Tension* was constant in the mind … on empty stomach the whole day … my head used to spin and go ‘boom,’ ‘boom.’ I didn’t know the meaning of sleep. I used to be up till 3-4 AM and wouldn’t wake up before noon. When there is *tension* many kinds of *tensions* are formed … if I have some *tension* … much more *tension* comes up.” – Respondent-1 (GHQ-12 score: 11).

Another participant shared his experience of anhedonia, which was reported by the vast majority of this class:

“Yes, I feel that I haven’t been able to do anything for my family (financially) – that’s why I feel like a failure to myself. I had *tension* for two months after I got fired from the job. I didn’t feel good doing anything – didn’t feel peaceful standing somewhere, nor felt peaceful while eating, nor when I was sitting somewhere or when I was sleeping. If something good happens, it feels poisoned, I can’t enjoy it. Nothing feels good. This is how I’m feeling even right now.” – Respondent-9 (GHQ-12 score: 13).

Most respondents in Class-1 expressed experiencing thoughts of suicide or wanting their life to end due to difficulties in life. A few actually had attempted suicide or engaged in self-harm. Most, however, did not plan to commit suicide, but said that these thoughts were often present in their minds. One respondent shared:

“Yes, I feel that I can’t help my family (financially). I want to physically hurt myself. When my friends made fun of me, I cut myself with a knife. My father called me *nishkamla* … meaning I am a ‘good-for-nothing.’ I felt so hurt that my own father would call me such names. He said: ‘This imbecile is less useful than a cow!’ It was so disrespectful that I felt like crying. I bought four sleeping pills and mixed them with my tea and drank. If my father speaks like this to me it’s better to kill myself.” – Respondent-22 (GHQ-12 score: 22).

## Discussion

### Applicability of latent class analysis to inform the staged model of depression

The results suggest that LCA could be a suitable method to inform the staged model of depression from mild distress when confronted with problems, to higher levels of distress and difficulty facing problems, and to a third class characterized by high distress plus a host of depression symptoms, such as anhedonia and poor concentration. This extrapolation of the staged model via LCA is supported by previous findings as most latent class solutions in the literature are similarly bookended by a mild and severe class on either end, with moderate symptoms in the middle, consolidated in one or more classes. As LCA results are specific to the population under study, it can incorporate cultural variability of symptoms, and inform where groups of individuals are best suited to seek services, or what interventions should be offered to each group, in each unique context. Health promotion and preventive measures can be offered to the ‘wellness’ class with mild or no symptoms. Members of the ‘distressed’ class, which had the most heterogenous experiences as captured by the qualitative research, may be most likely to transition up or down in the distress continuum. Accordingly, these individuals could be directed to community-based interventions, or psychotherapeutic interventions by non-specialists, and referrals if necessary. The ‘severely distressed’ class indicate those at highest risk, or those who are potentially already disordered, and can be referred to the health system and specialist care for assessment and appropriate therapeutic or pharmacological interventions. Our findings could not derive a ‘refractory’ stage, as indicated in the staged model, but perhaps, such an impaired state can only be determined by repeated assessment over long periods. Individuals with refractory syndromes would likely be members of the ‘severely distressed’ class.

### A distress-continuum, disorder-threshold model of depression

It is interesting that not all symptoms of the GHQ-12 fall along a continuum. Rather, a cluster around stress and strain, inability to face difficulties, and inability to take decisions, seems to fall along a severity continuum, across the three derived classes. These symptoms were the only areas of marked difference between the ‘distressed’ and ‘wellness’ classes. Hallmark symptoms like anhedonia, depressed mood, and cognitive impairment were not on a continuum, and only emerged for the ‘severely distressed’ class. This may be, in some ways, a potential parallel to a physical illness, such as Type II diabetes mellitus. There is a continuum of mild to moderate obesity (continuum) that typically precedes diabetes, then once an individual has passed a threshold there is glucose dysregulation and a host of diabetic sequelae (threshold effect), e.g., retinopathy, neuropathy, and nephropathy. Similarly, there may be a continuum of feeling under strain and difficulty managing problems, but after a certain level of feeling under strain, this may transform into anhedonia and the host of depression symptoms.

This pattern raises questions about the entire range of depressive symptoms being on a continuum and may be better explained by the idea of ‘central symptoms’ from network analysis of psychopathology [51]. This approach suggests that certain symptoms, which are centrally located in networks of symptoms (derived by the number of connections with other symptoms, and/or the strength of the relationships between symptoms), when activated, are the causal impetus behind the emergence of other, more severe symptoms. These key symptoms of strain, indecisiveness, and inability to face problems, when increasing in severity, may be driving the manifestation of more severe and hallmark symptoms of depressive disorder and warrant future research with network analysis methods to examine the role of centrality in psychopathology further.

This continuum of strain, indecisiveness, and inability to overcome difficulty, seems to align with the local use of the *tension* idiom to communicate a range of symptoms of increasing intensity. Likely this *tension* cluster, when increasing in severity, may be driving the emergence of more severe and hallmark symptoms of depressive disorder. The study findings indicate that the *tension* cluster is where preventive measures may be most effective to prevent progression into full blown disorder. The local construct of *tension* needs to be studied in future research to determine its meaning across the range of severity, and its perceived causes and implications, to inform locally suitable intervention efforts.

**Fig. 2:**
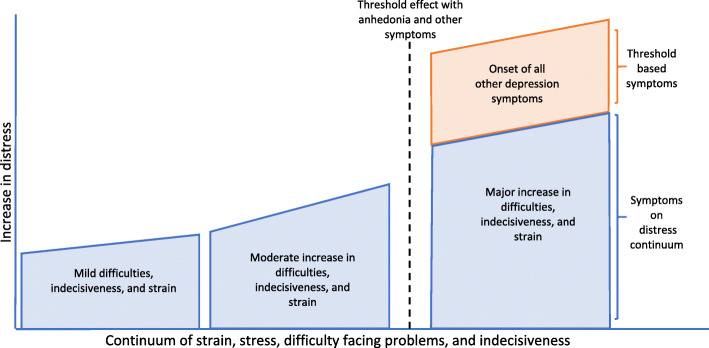
A distress-continuum, disorder-threshold model of depression

Accordingly, from our findings we propose a ‘distress-continuum, disorder-threshold’ model (Fig. 2) of depression. This model needs to be tested in different populations and contexts using LCA and other methods like network analysis to see if similar patterns can be detected. We recommend the inclusion of both distress and depressive symptoms in future analyses, as scales with disorder symptoms alone would theoretically fail to capture this pattern. The GHQ-12 would be an ideal scale for this purpose. Additionally, a social epidemiological analysis of the stress-distress continuum examining correlates to the latent classes in a larger and more diverse population with a wider spectrum of risks would be a valuable direction for future research as well.

### Strengths and limitations

This current study has a few limitations. First, although latent class analysis can be used to derive classes along a continuum of distress allowing policy decision-making on how to direct each class to appropriate services, the analysis does not perfectly derive the four tiers of the staged model, and likely will require individual assessment by specialists for those at the severe end to screen for refractory cases. Secondly, qualitative research with young men from a culture dominated by norms of masculine strength and infallibility may have restricted full disclosure during data collection. Finally, we identified a few negative cases in the qualitative analyses that did not align with the quantitative findings. As the qualitative interviews were conducted 1.5 years after the quantitative survey, some respondents’ mental state could have changed over time, possibly explaining the discrepancy.

Regardless of the limitations, the current study has some major strengths and addresses two critical gaps in the literature by putting forward an empirical approach to informing the staged model of depression and providing a detailed analysis of distress and depressive experiences of slum dwelling young men, a largely understudied, yet highly vulnerable population in global mental health. The use of qualitative interviews allowed deeper insight into the classes, examining the context and causes behind the experiences described by the respondents. This study also offers a more nuanced interpretation of the continuum of distress, and provides preliminary evidence warranting further research into depression psychopathology around the world.

## Conclusions

The findings of this study provide support for latent class analysis as a useful empirical tool to inform a staged model of depression. The results also contribute to the literature by suggesting a progression of distress through to disorder along a distress continuum, wherein a constellation of impairing symptoms emerge together after exceeding a high level of distress, i.e., a tipping point of *tension* heralds a host of depression symptoms. Future research is necessary to establish if such a continuum of distress precedes disorder in various populations across multiple contexts.

## Data Availability

The datasets for this study are available from the corresponding author on reasonable request.
